# Detection of circular RNA expression and related quantitative trait loci in the human dorsolateral prefrontal cortex

**DOI:** 10.1186/s13059-019-1701-8

**Published:** 2019-05-20

**Authors:** Zelin Liu, Yuan Ran, Changyu Tao, Sichen Li, Jian Chen, Ence Yang

**Affiliations:** 1 Institute of Systems Biomedicine, Department of Medical Bioinformatics, School of Basic Medical Sciences, Peking University Health Science Center, Beijing, 100191 People’s Republic of China; 2 Institute of Functional Nano and Soft Materials (FUNSOM) and Collaborative Innovation Center of Suzhou Nano Science and Technology, Soochow University, Suzhou, 215123 People’s Republic of China; 3 Department of Microbiology and Infectious Disease Center, School of Basic Medical Sciences, Peking University Health Science Center, Beijing, 100191 People’s Republic of China; 4 Department of Industrial Engineering and Operations Research, School of Engineering and Applied Science, Columbia University, New York, NY 10027 USA; 5 Key Laboratory of Neuroscience (Peking University), Ministry of Education, Beijing, 100191 People’s Republic of China

**Keywords:** Circular RNA, circQTLs, Expression variation, GWAS

## Abstract

**Background:**

Circular RNAs (circRNAs) are implicated in various biological processes. As a layer of the gene regulatory network, circRNA expression is also an intermediate phenotype bridging genetic variation and phenotypic changes. Thus, analyzing circRNA expression variation will shed light on molecular fundamentals of complex traits and diseases.

**Results:**

We systematically characterize 10,559 high-quality circRNAs in 589 human dorsolateral prefrontal cortex samples. We identify biological and technical factors contributing to expression heterogeneity associated with the expression levels of many circRNAs, including the well-known circRNA CDR1as. Combining the expression levels of circRNAs with genetic *cis*-acting SNPs, we detect 196,255 circRNA quantitative trait loci (circQTLs). By characterizing circQTL SNPs, we find that partial circQTL SNPs might influence circRNA formation by altering the canonical splicing site or the reverse complementary sequence match. Additionally, we find that a subset of these circQTL SNPs is highly linked to genome-wide association study signals of complex diseases, especially schizophrenia, inflammatory bowel disease, and type II diabetes mellitus.

**Conclusions:**

Our results reveal technical, biological, and genetic factors affecting circRNA expression variation among individuals, which lead to further understanding of circRNA regulation and thus of the genetic architecture of complex traits or diseases.

**Electronic supplementary material:**

The online version of this article (10.1186/s13059-019-1701-8) contains supplementary material, which is available to authorized users.

## Background

Circular RNAs (circRNAs) are a class of recently identified long noncoding RNAs with a covalently closed continuous loop structure formed via back-splicing that have neither a 5′ cap nor a 3′ polyadenylated tail [1]. Thousands of circRNAs have been identified in humans and other species [2]. A handful of circRNAs have been functionally elucidated, and these functions include acting as sponges for microRNAs [3, 4], competing for RNA-binding protein [5], and even translating into protein [6, 7]. circRNAs are thought to play crucial roles in multiple cellular processes and disease pathogenesis based on their tissue- and development-specific manner of expression [3, 4, 8]. As a layer of the gene regulatory network, circRNA expression is also an intermediate phenotype bridging genetic variants and phenotypic changes. Thus, understanding circRNA expression variation will shed light on the molecular fundamentals of complex traits and diseases.

However, due to the intrinsic circRNA characteristic of lacking a polyadenylated tail, circRNA expression has been underestimated by RNA-seq using the polyadenylated selection method. Several studies have identified circRNAs and quantified their abundance at the transcript level [9, 10], but small sample sizes limited their efforts to systemically dissect changes in circRNA expression. Data from the CommonMind Consortium (CMC) provide an opportunity to decipher circRNA expression variation. The CMC comprises nearly 600 brain samples obtained from the autopsies of individuals with and without severe psychiatric disorders [11]. Data generated from the CMC include SNP genotypes and gene expression (RNA-seq) as well as other functional genomic data. More importantly, CMC RNA sequencing libraries were prepared by ribosomal RNA depletion, which facilitated the analysis of circRNA expression.

In the current study, we utilized genotype and dorsolateral prefrontal cortex (DLPFC) expression data provided by the CMC to exploit circRNA expression variation arising from genetic, biological, and technical factors. We detected more than 10,000 high-quality circRNAs in the samples using stringent methodologies. We then highlighted the effects of covariates on circRNA expression variation. By circQTL analysis, we characterized the *cis*-regulation of circRNA and found that circQTL SNPs were enriched among disease risk loci. Our findings will further our understanding of the genetic architectures of complex traits or diseases.

## Results

### Identification of circRNAs in the human brain

**Fig. 1:**
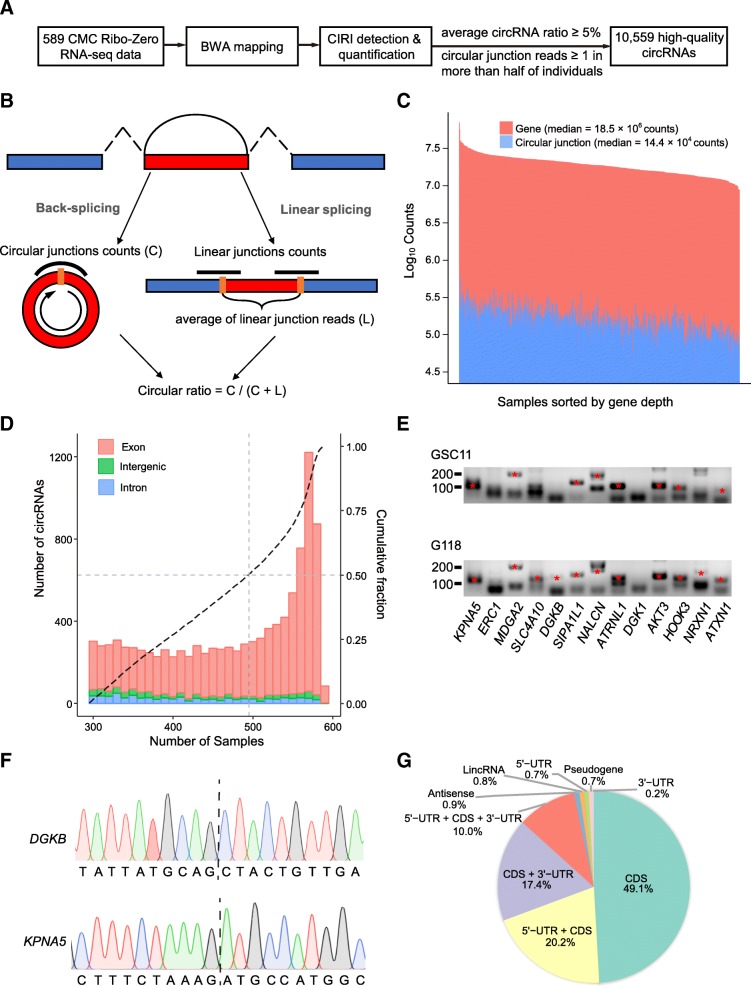
Profiling of circRNAs in the DLPFCs of 589 individuals. **a** The analysis pipeline to identify and quantify high-quality circRNAs. **b** A schematic diagram for calculating the circular ratio. **c** The stack diagram showing distributions of total gene counts and potential circular junction counts. The counts are log base 10 transformed on the *y*-axis. **d** The sample distribution for existing circRNAs in exonic, intronic, and intergenic regions. The black dashed line indicates the cumulative data fraction. **e** Gel analysis shows the target RT-PCR product bands. Red star points indicate the target band. **f** Two Sanger sequencing examples showed that RT-PCR products spanned the circular junction. Dashed line indicates the circular junction site. **g** Annotations of genomic regions mapping to inferred exonic circRNAs. CDS: coding sequence; lincRNA: long intergenic noncoding RNA; UTR: untranslated region

We systemically identified circRNAs by analyzing Ribo-Zero RNA-seq data from postmortem DLPFC samples collected from 258 schizophrenia (SCZ) patients, 54 affective/mood disorder (AFF) patients, and 277 controls (Table 1) downloaded from the CMC database [11]. We first de novo identified circRNAs originating from canonical GT-AG splicing using CIRI [12, 13] (Fig. 1a), which achieved better balanced performance between precision and sensitivity [14] and reported both circular junction counts and the circular ratio (Fig. 1b). By using a loose criterion (circular junction counts ≥ 1), we detected 9776 to 63,781 (median 31,286) potential back-splicing sites and 30,188 to 577,672 (median 143,573) potential circular junction counts (Fig. 1c) for each sample. These junction counts account for average about 0.8% of the total gene counts, which are higher than most of other tissues and cell lines (Additional file 1: Table S1; Additional file 2: Figure S1). Then, we screened out circRNAs expressed in the human brain (circular junction counts ≥ 1 in more than half of individuals) with a potential function instead of byproducts of linear splicing (average circular ratio ≥ 0.05). Finally, we identified 10,559 high-quality circRNAs (Additional file 3: Table S2), half of which were detected in 495 samples at least (Fig. 1d), and 781 (7.4%) of which expressed higher than their linear counterparts (circRNA ratio > 0.5).About 99.7% (10,526 of 10,559) predicted high-quality circRNAs could also be detected by find_circ [4] and/or CIRCexplorer [15] (Additional file 3: Table S2). Approximately 11.6% (1229 of 10,559) of the high-quality circRNAs were not mentioned in previous studies [2, 9]. Among them, 13 highly expressed circRNAs were detected in two human glioma stem cell lines (GSC11 and G118, details in the “Materials and methods” section) [16]. And 11 of 13 (85%) back-splicing events were experimentally validated by two rounds RT-PCR with divergent primers (Fig. 1e; Additional file 4: Table S3). Then, we further confirmed our analysis with five randomly selected products by Sanger sequencing (Fig. 1f; Additional file 2: Figure S2). Together, these results supported the existence of 1229 novel circRNAs in CMC dataset.

**Table 1:** Sample description

Variables	Controls	SCZ	AFF
Subjects	277	258	54
Male sex, *N* (%)	158 (57.0)	164 (63.6)	27 (50.0)
Caucasian ethnicity^a^, *N* (%)	205 (74.0)	209 (81.0)	51 (94.4)
Age of death^b^ (years), mean (SD)	65.1 (18.9)	68.7 (16.5)	51.5 (16.1)
Postmortem interval (h), mean (SD)	13.6 (7.9)	20.8 (13.5)	19.3 (7.1)
RNA integrity number, mean (SD)	7.8 (0.8)	7.4 (0.9)	7.9 (0.7)
Gene aligned reads (10^6^), mean (SD)	20.1 (6.5)	17.7 (5.7)	21.0 (4.5)
Linear junction aligned reads (10^5^), mean (SD)	57.2 (32.6)	47.7 (26.7)	56.4 (24.0)
Circular junction aligned reads (10^4^), mean (SD)	16.9 (7.3)	15.0 (6.7)	15.3 (5.9)

^a^Genetic inferred Caucasian ethnicity

^b^Age of death ≥ 90 was considered 90 due to the limited information in the original data source

Consistent with a previous study on circRNAs in HEK293 cells [4], 87.1% of the circRNAs (9198 of 10,559) were located in exonic regions, followed by intronic (7.0%) and intergenic (5.9%) regions in the human brain. Most (96.7%) exonic circRNAs overlapped with the coding sequence region (Fig. 1g) according to the GENCODE annotation. The median number of exons and median length of exonic circRNAs were determined to be five and 780 bp, respectively (Additional file 2: Figure S3).

The 9935 exonic and intronic circRNAs were derived from 3916 genes, 45.9% of which generated only one circRNA (Additional file 2: Figure S4). To analyze the functions of circRNA host genes, the 3916 host genes were uploaded to DAVID tools [17] (https://david.ncifcrf.gov/) as a test gene list, and 16,423 expressed genes from the same CMC sample set [11] served as the background. Alternative splicing (Benjamini-corrected *P* = 4.99 × 10^−78^, Fisher’s exact test (FET)) and splice variants (Benjamini-corrected *P* = 3.65 × 10^−65^, FET) were the most significantly enriched among the host genes (Additional file 5: Table S4), consistent with the existing knowledge that back-splicing is correlated with linear splicing [18]. A previous study observed that circRNAs are enriched in synapses in the mouse brain [19]. Interestingly, postsynaptic membrane (Benjamini-corrected *P* = 0.0083, FET) and postsynaptic density (Benjamini-corrected *P* = 0.039, FET) were enriched in our detected host genes, which also suggests that circRNAs play important roles in synaptic function.

### Normalization of circRNA expression and evaluation of covariate effects

**Fig. 2:**
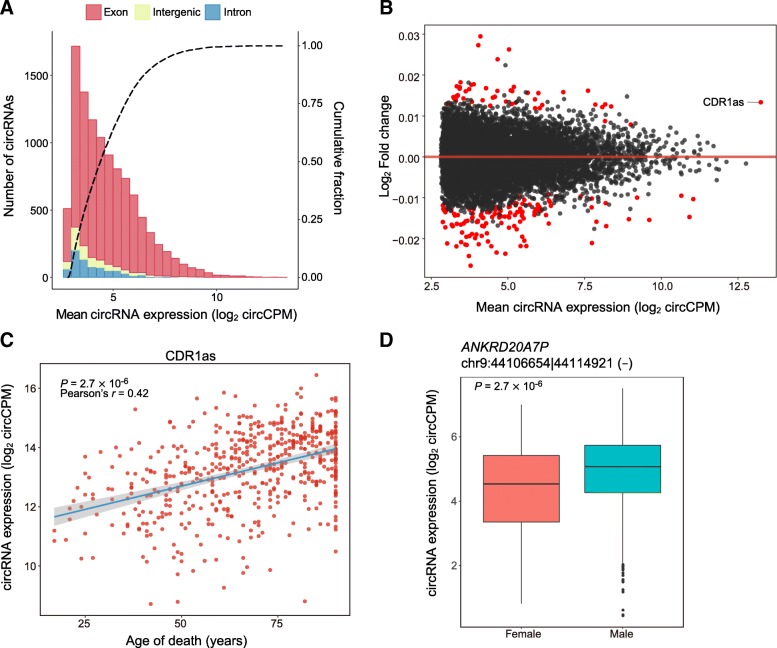
Effects of covariates on circRNA expression. **a** Distribution of the mean expression of each circRNA in exonic, intronic, and intergenic regions. The black dashed line indicates the cumulative data fraction. **b** Differential circRNA expression detected as a function of age of death. The dots in red indicate significantly differentially expressed circRNAs (FDR ≤ 0.05). **c** CDR1as expression with age of death is plotted. **d** Boxplot for the expression of gender-related circRNA (chr9:44106654|44114921) from a pseudogene, *ANKRD20A7P*. The center line denotes the median, the limits are the interquartile range (IQR), the whiskers are 1.5× the IQR, and outliers are shown as black dots

Considering the stability differences between circular and linear RNAs, we measured the circRNA expression levels with voom [20] by **circ**ular junction **c**ounts **p**er **m**illion circular junction reads (circCPM), which normalized the sequencing depths among samples (Fig. 2a). Similar to a previous study [11], both the technical (institution, library preparation batch (LIB), postmortem interval (PMI), RNA integrity number (RIN)) and the biological (age of death (AOD), ethnicity, gender, SCZ) effects were considered as covariates. We applied the linear regression utilities in the limma [21] package to evaluate the effects of these factors on each circRNA. In contrast, we used the same method to analyze the effects of covariates on gene expression normalized by **g**ene **c**ounts **p**er **m**illion gene reads (gCPM; see the “Materials and methods” section). Compared to linear RNAs, the expression of circRNAs is less affected by all the covariates than linear RNAs (Table 2).

**Table 2:** The numbers of circRNAs and genes affected by technical and biological factors at an FDR < 0.05

Type	Institution	LIB	PMI	RIN	AOD	Ethnicity	Gender	SCZ
circRNA (*N* = 10,559)	300 (2.8%)	4143 (39%)	2 (0.02%)	28 (0.27%)	175 (1.7%)	249 (2.4%)	35 (0.33%)	0 (0%)
Gene (*N* = 16,422)	13,291 (81%)	14,759 (90%)	4033 (24%)	11,121 (68%)	9668 (59%)	1097 (6.7%)	226 (1.4%)	926 (5.6%)

The library batch is the most significant factor impacting both circRNA expression and gene expression. Compared to random sampling, LIB-associated circRNAs have higher expression levels (*P* < 1 × 10^−4^, bootstrapping) and shorter median lengths (*P* < 1 × 10^−4^, bootstrapping; Additional file 2: Figure S5), suggesting that the length of a circRNA may be related to circRNA stabilization. A tiny fraction of circRNAs was expressed in association with institution (2.8%), PMI (0.02%), and RIN (0.27%), consistent with circRNAs being more stable than linear RNAs.

For age of death, 49 circRNAs exhibited increased expression and 126 circRNAs showed decreased expression with age (Fig. 2b; Additional file 6: Table S5). Among 123 circRNAs originating from an explicit host gene, 32 are AOD-related, while their host genes are not, suggesting that the roles of these circRNAs in aging are independent of their counterparts. Interestingly, a famous brain-enriched circRNA, CDR1as, which is known as a sponge for miR-7 regulating various diseases, such as cancer [22] and neurodegenerative diseases [23], showed increased expression with age (Fig. 2c), implying that CDR1as may be involved in aging of the brain.

For gender, 21 of 35 differential circRNAs were located on sex chromosomes. After removing circRNAs from the sex chromosomes and readjusting the *P* values of the remaining circRNAs, we found only one circRNA that was differentially expressed (DE). This circRNA was highly expressed in males and originated from the pseudogene *ANKRD20A7P* (Fig. 2d), which is especially highly expressed in testes [24].

For schizophrenia, we did not detect any DE circRNAs after false discovery rate (FDR) correction (top FDR = 0.53, *P* = 1.1 × 10^−4^) between cases and controls, or randomly selected samples (100 *vs*. 100, 50 *vs*. 50) for 1000 times (see details in the “Materials and methods” section). Although gender differences exist in susceptibility of SCZ [25], neither male-specific nor female-specific DE circRNAs were detected. In the previous analysis, we filtered out circRNAs in a sample set including both SCZ and control populations. To further mine disease-specific expressed circRNAs, we reanalyzed 303 circRNAs that were expressed in either SCZ or control populations. With the same procedure, we still failed to detect any DE circRNAs (see details in the “Materials and methods” section). Considering that there are hundreds of DE genes but no DE circRNAs, we compared the distributions of effect size between genes and circRNAs and found that the effect size of circRNAs is smaller than genes (Additional file 2: Figure S6). Thus, it requires 275 cases *vs*. 275 controls to detect DE genes, but 435 cases *vs*. 435 controls to detect DE circRNAs, at the significance level of 80% power (Bonferroni-corrected *P* < 0.05, Student’s *t* test; see details in the “Materials and methods” section). As there are only 258 SCZ and 277 control samples in CMC dataset, a larger sample size or more sensitive statistical methods may be required to detect DE circRNAs between SCZ and control.

### Effects of genetic variation on circRNA expression

To measure how circRNA expression is regulated by genetic variations, we analyzed the association of covariate-adjusted circRNA expression with genetic variants, i.e., circRNA quantitative trait loci (circQTLs). Similar to *cis*-sQTLs for alternative splicing [26, 27], we selected circRNAs in combination with high-quality (imputation quality score ≥ 0.8) and common SNPs (minor allele frequency (MAF) ≥ 0.05) in the ± 100-kb region of back-splicing sites to identify *cis*-circQTLs by using Matrix eQTL [28]. After permutation and *q* value [29] correction to control the FDR, we identified 251,374 circQTLs associated with 2790 circRNAs.

**Fig. 3:**
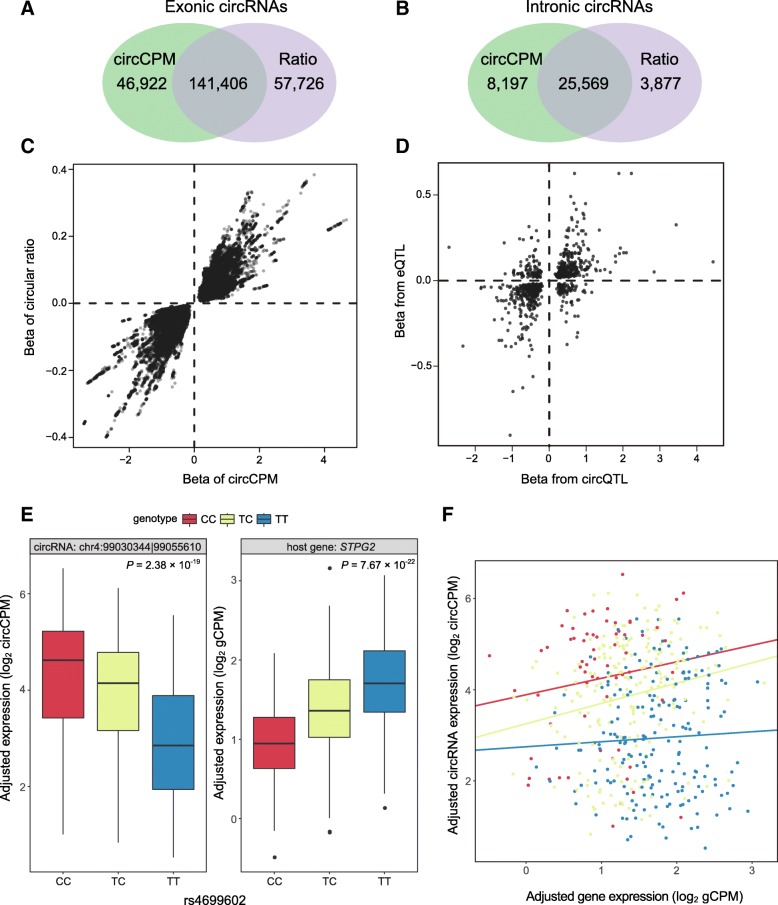
Identification of *cis*-circQTLs. **a**, **b** circQTLs detected by circCPM and the circular ratio for exonic (**a**) and intronic (**b**) circRNAs. **c** The concordant effects of circQTLs detected by the circCPM and circular ratio methods. **d** The effects of circQTLs *vs*. eQTLs. **e**, **f** One example of a discordant QTL between a circRNA and its host gene. The center line denotes the median, the limits are the interquartile range (IQR), the whiskers are 1.5× the IQR, and outliers are shown as black dots. **f** The expression level of the circRNA and the gene in each sample. The red, yellow, and blue lines fit the expression between the gene and circRNA according to the CC, TC, and TT genotypes, respectively

A weak correlation has been reported between circRNAs and linear RNA expression [30, 31]. To exclude circQTL SNPs that regulate host gene expression rather than circRNA expression, we further refined the circQTL SNPs regulating exonic and intronic circRNAs by reanalyzing *cis*-circQTLs with a circular ratio instead of circCPM. Following the same pipeline, we detected 228,578 circQTLs, 166,975 (73.05%) of which were identified in both (Fig. 3a, b) and with concordant effects (Fig. 3c). In addition to the intergenic circQTLs, which lack host genes, we finally identified a total of 196,255 circQTLs associated with 2086 circRNAs involving 1269 genes (Additional file 7: Table S6).

To estimate the proportion of significant circQTLs that are also eQTLs, we selected the most significant circQTL SNP for each circRNA (max-circQTL) to characterize circQTLs. Compared with eQTLs identified in the same dataset [11], approximately 48.6% (851 of 1750) of max-circQTLs of circRNAs with an explicit host gene were also eQTLs. For SNPs that were both circQTLs and eQTLs, 77.9% (663 of 851) exhibited concordant effects (Fig. 3d), while the others were discordant. For the SNPs that are both eQTLs and circQTLs, we observed that there may be an interactive expression relationship between the circRNA and the host gene (Fig. 3e, f).

### Potential mechanisms of circQTL

**Fig. 4:**
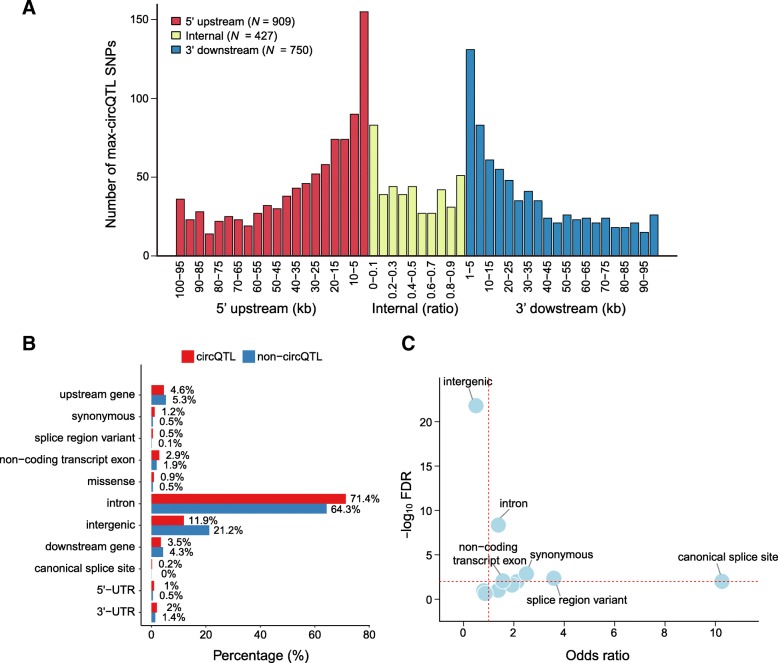
Distribution of the identified circQTL SNPs. **a** Distance distribution of the most strongly associated circQTL SNPs for each circRNA (max-circQTL) to their nearest back-spliced site. For internal max-circQTL SNPs, the distance was calculated by the ratio of the distance between the SNP and the 3′ to 5′ back-splicing site. **b** Comparison of the distributions of sequence-defined elements between pruning max-circQTL SNPs and non-circQTL SNPs. **c** Enrichment of max-circQTL SNPs among sequence-defined elements. The dashed horizontal line indicates an FDR = 0.01, and the dashed vertical line indicates an odds ratio (OR) = 1, two-tailed FET

To study the potential mechanisms of circQTLs, we first analyzed the distance distribution of max-circQTL SNPs to the back-splicing site. We found that the max-circQTLs were preferentially located in proximity to the back-splicing acceptor or donor sites. About 22.1% (462 of 2086) max-circQTL SNPs were located in flanking intron of junction sites and 20.5% (427 of 2086) were located in circRNA region. These results suggested that SNPs located in flanking sequences more likely contribute to circRNA regulation (Fig. 4a).

We tested the enrichment of 1721 linkage disequilibrium (LD)-based pruning max-circQTL SNPs against sequence-defined elements by using the two-tailed FET; the MAF and distance-matched non-circQTL SNPs served as controls (see the “Materials and methods” section). We found that intron was the most enriched (Fig. 4b, c; FDR = 4.31 × 10^−3^, OR = 3.59, two-tailed FET) among 11 sequence-defined elements, which suggested that introns are implicated in circRNA circularization, as noted in previous studies [32]. A previous study reported that reverse complementary sequences (RCSs) in introns promote circRNA formation [33]. We also found that 1229 pruning max-circQTL SNPs located in introns were enriched in RCSs (11.5%, *P* = 1.26 × 10^−4^, OR = 1.45, two-tailed FET), with non-circQTL SNPs in introns serving as controls.

**Fig. 5:**
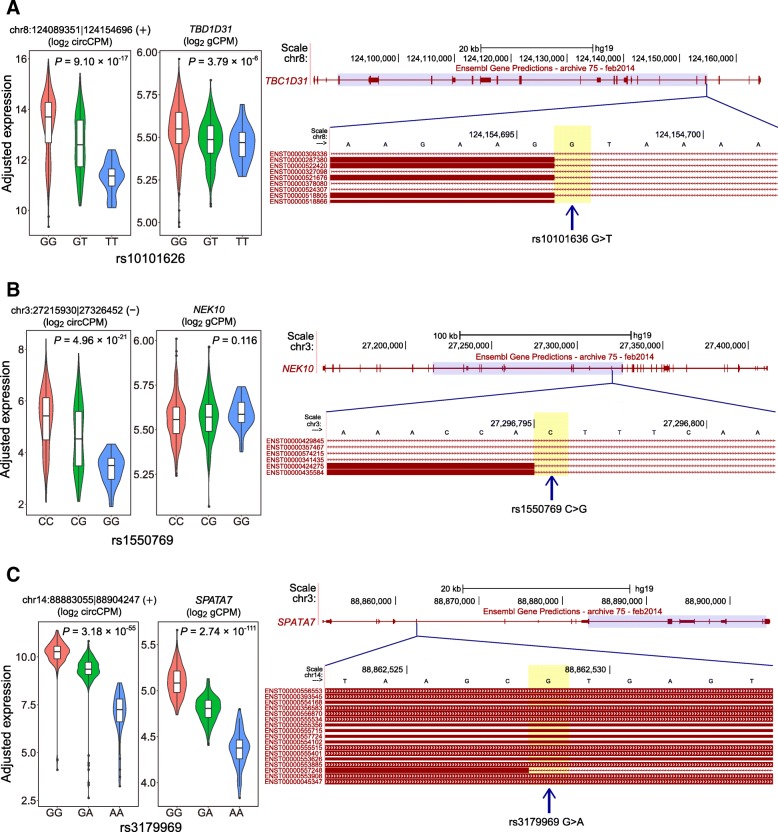
Impacts of the canonical splice site on circRNA expression. Violin plots of covariate-adjusted circRNA and gene expression within each genotype are shown in the left panels. The overlaid boxplots indicate the median (horizontal black lines) and interquartile range (IQR, white boxes). Outliers are shown as black dots. Schematic of transcript isoforms at each locus (Ensembl Gene Predictions tracks from the UCSC Genome Browser) are shown in the right panels. The back-splicing region is highlighted in purple. Arrowheads indicate the splice sites. **a** circQTL SNP at the canonical back-splicing site in *TBC1D31*. **b** circQTL SNP at the circRNA internal canonical splice site in *NEK10*. **c** circQTL SNP at the circRNA external canonical splice site in *SPATA7*

Canonical back-splicing signals are required for circularization [18]. Thus, we manually inspected SNPs located in back-splicing sites from all 1,611,445 SNPs for circQTL identification. We detected seven canonical back-splicing SNPs (Additional file 8: Table S7), all of which were identified as circQTL SNPs. The circRNA expression of individuals with the canonical splice genotype was higher than that with the non-canonical splice genotype (Fig. 5a) in all seven SNPs. Four of the seven back-splicing SNPs were also significantly related to their host gene expression with three concordant and one discordant effects [11]. Our results supported that circQTL SNPs located in canonical back-splicing sites may influence circRNA expression by disrupting circRNA formation.

Among the 11 sequence-defined elements, the canonical splice site had the highest OR (Fig. 4c; FDR = 9.73 × 10^−3^, OR = 10.3, two-tailed FET). We manually detected 17 SNPs (Additional file 8: Table S7) at the canonical splicing site but not at the back-splicing site that were located in the host genes of regulated circRNAs. All the SNPs effected on the expression and/or splicing of their host genes. For the three circRNA with internal splice site (Fig. 5b), the expression levels were higher in the individuals with canonical splice genotype, implying that internal splicing of circRNA may promote circRNA formation or that circRNAs with retained introns are more likely to be degraded. While for the circRNAs with external splice site (Fig. 5c), the 14 circRNAs were not strictly associated with the canonical splice site, which indicated the complex effects of distant splicing on circRNA formation.

### Colocalization between circQTL SNPs and disease risk loci

Thousands of genetic loci harboring disease- and trait-associated variants have been identified by GWAS [34]. However, it is often unclear how the genotype is related to the phenotype. Similar to other QTL analyses, i.e., eQTL [11] and sQTL [26], circQTL may also be helpful for understanding the mechanisms underlying the risk of genetic variants identified by GWAS. Thus, we performed enrichment analysis for pruning max-circQTLs using data from the GWAS Catalog [34], a collection of data from GWAS for various human diseases and traits.

**Fig. 6:**
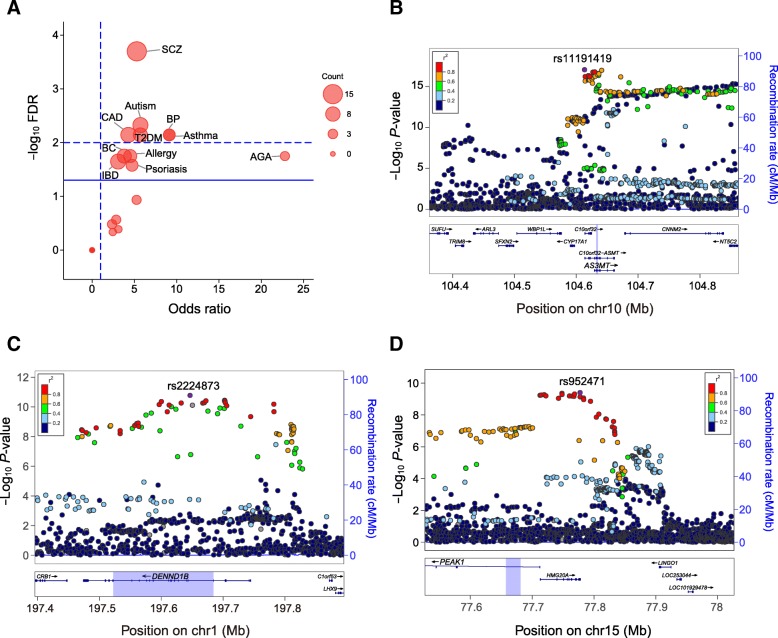
circQTL SNPs enriched in GWAS loci. **a** Enrichment of pruning max-circQTL SNPs among the GWAS Catalog. The circle size is proportional to the number of overlapping SNPs between GWAS loci and pruning max-circQTLs. The dashed horizontal line indicates an FDR = 0.01, the solid horizontal line indicates an FDR = 0.05, and the dashed vertical line indicates an OR = 1, one-tailed FET. AGA: androgenetic alopecia; BC: breast carcinoma; BP: bipolar disorder; CAD: coronary artery disease; IBD: inflammatory bowel disease; T2DM: type II diabetes mellitus. **b**–**d** Local plots of circQTL SNPs with high linkage disequilibrium (*r*^2^ > 0.8) with GWAS results of **b** schizophrenia [35], **c** inflammatory bowel disease [36], and **d** type II diabetes mellitus [37] were generated by LocusZoom [38]. The most significant GWAS SNPs are colored purple. Nearby SNPs are color-coded according to their LD (*r*^2^). The back-splicing region is highlighted in purple. The statistical strength of the association (− log_10_ *P* values) and recombination rate are double-plotted on the *y*-axis. Genes in the UCSC Genome Browser are shown in the panels below the local plots

We found that pruning max-circQTL SNPs were significantly enriched among loci associated with diseases compared with non-circQTL SNPs (*P* = 3.92 × 10^−17^, OR = 3.53, one-tailed FET). We further analyzed the enrichment using the data of 21 individual diseases with risk loci > 90 in the catalog (see “Materials and methods” section) and found significant enrichment of circQTL SNPs among loci associated with 11 diseases (Fig. 5a). Because the CMC dataset derives from the DLPFC of SCZ patients and controls, SCZ-associated loci were the most enriched (Fig. 6a; FDR = 2.00 × 10^−5^, OR = 5.28, one-tailed FET), as expected.

By testing all circQTL SNPs in high linkage disequilibrium (*r*^2^ > 0.8) with GWAS SNPs (Additional file 9: Table S8), we further identified 157 circRNAs associated with 122 diseases (one circRNA can be involved in multiple diseases). For example, we observed 40, 35, and 21 circRNAs associated with SCZ, inflammatory bowel disease, and type II diabetes mellitus, respectively (Fig. 6b–d). Interestingly, an SCZ-related linear isoform *AS3MT*^d2d3^ [39] was also regulated by the SCZ risk loci (Fig. 6b), implying a potential mechanism between the linear and circular isoform regulated by SCZ risk loci. Notably, 72.6% (114 of 157) circRNAs could be regulated by GWAS-linked circQTL SNPs located in flanking introns and circRNA regions, which also suggested the important roles of flanking introns and circRNA regions in pathogenesis. The colocalization between circQTL and GWAS loci provides novel insights into the pathogenesis of complex diseases.

## Discussion

Thousands of genetic variants have been associated with diseases by GWAS. However, how these variants exert their effects on diseases remains largely unknown. QTL analysis is a powerful tool for understanding the mechanisms underlying these genetic variants because the target gene (or DNA functional element) via which a genetic variant leads to disease can be identified by determining whether a risk variant also regulates a transcriptomic quantitative trait, such as the eQTL and sQTL [40, 41]. As a layer of the gene regulatory network, circRNA expression variation may underlie the mechanisms of risk loci. Thus, we introduced circQTLs, which represent the expression levels of circRNAs regulated by genetic variants, to unravel the mechanism of GWAS SNPs. We found that circQTL SNPs were significantly enriched for the GWAS variants associated with various diseases, such as schizophrenia, inflammatory bowel disease, and type II diabetes mellitus. With our systematic analysis of genetically mediated circRNA expression, we show that circQTLs could be used to refine the functional consequences of GWAS loci as another QTL approach. We also tried to identify potential mechanisms of circQTLs and found variants at canonical back-splicing sites in response to lower circRNA expression, implying that canonical splicing signals are required for circularization. In addition to back-splicing sites, the internal and external splicing sites of a circRNA may contribute to its expression. In addition, we showed that circQTLs were enriched in RCSs, which play important roles in exon circularization [33]. Although we proposed several potential mechanisms, they may explain less than 10% of circQTLs. Further studies are needed to unravel the mechanisms underlying these circQTLs.

One challenge in analyzing circQTL is to partition circRNA expression from that of its host gene. Here, we identified circQTLs by integrating two methods, the circular junction and circular ratio, to filter out false positives, which are actually eQTLs of the counterpart. Each approach has strengths and weaknesses that complement each other. The advantage of the circular junction approach over the ratio approach is that it detects real circRNA expression levels that play functional roles, while the ratio approach detects only circRNA expression relative to its linear expression. However, the circular junction approach cannot distinguish whether an SNP influences the expression of the host gene or the circRNA itself, while the advantage of the ratio is that the relative value can represent the splicing condition, which directly reflects circRNA formation. Although these two approaches use different quantization strategies, more than 70% of circQTLs were detected in both approaches with concordant effects.

In addition to genetic factors, i.e., circQTLs, biological and technical factors may also contribute to circRNA expression variation. However, circRNAs are less affected by these factors, mainly because circRNAs are more stable than linear RNAs. The library batch influenced 39% of circRNAs, indicating that it is a key factor that should be considered in circRNA studies, especially for differential expression analysis. We identified a few circRNAs associated with SCZ (0), PMI (2), and gender (39), but 175 AOD-related circRNAs were identified, among which 44 circRNAs were shown to be AOD-related independent of their host gene or located in the intergenic region. These circRNAs include CDR1as, which is highly expressed in the human brain [42], chr1:66378928|66384518 from *PDE4B*, which interacts with *DISC1* to regulate cAMP signaling in schizophrenia [43], and chr2:160193973:160252345 from *BAZ2B*, which belongs to the bromodomain gene family associated with transcriptional activation [44]. Considering that circRNAs accumulate in the brain with increasing age [45], these age-related circRNAs may provide new insights into understanding the aging of the human brain or aging diseases.

Dozens of SCZ GWAS risk loci, identified from a large cohort (36,989 cases *vs*. 113,075 controls) [35], are found to be implicated in circRNA expression, but none of these SCZ GWAS risk circRNAs (circRNA regulated by SCZ GWAS risk loci in circQTL relation) were DE between SCZ and controls in the CMC dataset. Previous study [11] has also encountered this “contradict” in linear gene expression, i.e., no DE evidence was found for SCZ GWAS risk genes (genes regulated by SCZ GWAS risk loci in eQTL relation). For detecting differential expression of SCZ GWAS risk genes at the power of 80%, the median number of subjects with SCZ and controls was estimated to be 28,500 [11]. Similarly, tens of thousands of samples are also needed for detecting differential expression of SCZ GWAS risk circRNAs, considering the small difference of risk allele frequencies in CMC dataset.

## Conclusions

In summary, our work identified and quantified thousands of circRNAs across hundreds of individuals and focused on the variation of circRNA expression. Unlike linear RNA, circRNA is impacted less by technical and biological effects. Although we detected some circRNAs related to age, mechanistic studies driven by the proposed circRNA candidates will be informative for aging. By performing circQTL analysis, we extended the genetic architecture of disease to circular RNAs. How circRNAs impact the pathogenesis of disease remains to be answered, but our results strongly suggest that circRNAs contribute to disease risk.

## Materials and methods

### Dataset description

In total, 589 individuals from the CMC database, including 258 SCZ patients, 54 affective/mood disorder patients, and 277 controls, were utilized in this study. Imputation genotyping data (imputed with IMPUTE2 [46]) of autosomes (Synapse: syn3275221) and RNA-seq data (BAM files), including mapped and unmapped reads, were downloaded (Synapse: syn4923029) using the Synapse command line client. Details regarding the RNA-seq analysis and data processing are available at the CMC wiki page (https://www.synapse.org/#!Synapse:syn2759792/wiki/69613). The mapped and unmapped reads of each sample were merged and converted into FASTQ format using SAMtools [47] (version 1.3.1).

### Detection of circRNA

The reads in the FASTQ files generated above were mapped onto the reference human genome (hg19) using the BWA [48] memory module with the parameter –T 19. CIRI [12, 13] (version 2.0.6) was used to detect putative circRNAs with the parameter −0 –A using the gene annotation file (GENCODE v19, http://www.gencodegenes.org) from each mapped file. CIRI calculates the number of circular junction counts and the circular ratio using the following formula:

$$ \mathrm{Circular}\ \mathrm{ratio}=\frac{C}{C+L} $$

where *C* represents the number of circular junction counts of a predicted circRNA and *L* represents the mean number of reads mapped across the two back-splicing sites but is consistent with linear RNA. circRNAs with circular junction counts ≥ 1 in more than half of the individuals were considered expressed. In addition, a candidate was eliminated if the average circular ratio < 0.05, which may be a result of linear splicing byproducts [10].

Besides CIRI, find_circ [4] and CIRCexplorer [15] were also used to identify back-splicing sites. For find_circ, raw reads of RNA-seq were mapped to the reference human genome and unmapped reads were used to detect circular junctions with default parameters [4]. For CIRCexplorer, raw reads of RNA-seq were mapped to the reference human genome by STAR [49] (detailed parameters can be accessed from Additional file 10) and chimeric junction file were parsed with default parameters [15].

### Human glioma stem cell culture

Human glioma stem cell line GSC11 [16] was a gift from Dr. Xing Su and human glioma stem cell G118 was derived from a grade IV glioma on the right temporal lobe in the first affiliated hospital of Soochow University. These cell lines are not listed in the database of commonly misidentified cell lines maintained by ICLAC. GSC11 was authenticated by carrying unique mutations in ATR and ATX genes, and G118 was carrying frameshift in NF1 gene. All the cell cultures performed in this study have been tested as negative for mycoplasma contamination. Both cell lines were cultured under DMEM-F12 medium with N2 and B27. Twenty nanograms/milligram of EGF and bFGF (Sigma) was supplemented to keep the cell from differentiation. The culture medium was replaced every other day. All the cell culture-related reagents were from Gibco unless otherwise indicated.

### Experimental validation of circRNA

Thirteen highly expressed circRNAs with annotated adjacent exons (length > 40) of back-splicing were selected for validation (Additional file 4: Table S3). The total RNA of glioma stem cells were extracted by TRIzol (Invitrogen) and reverse transcribed by PrimeScript RT Reagent Kit (Takara). With divergent primers (Additional file 4: Table S3) designed in the two adjacent exons, 50 ng of cDNA template was used for the first round of PCR and 1/50 of the product from the first round of PCR was used as template for the second round of PCR. The final products were analyzed using 2% agarose gel. The PCR products were further cloned to pUcm-T vector (Sangon) if the bands were clearly visible and the clones with inserts were sequenced by Sanger sequencing (Genewiz).

### Evaluate relative circRNA expression level in human cell lines and tissues

Human Ribo-Zero RNA-seq data of 14 cell lines and 29 tissues were collected from ENCODE Project Consortium [50] (Additional file 1: Table S1). Gene counts were quantified by STAR with annotation file GENCODE v19. circRNAs were detected by CIRI with the same parameters as described in method of detection of circRNA. For each sample, total circular junction counts were calculated by summing circular junction counts of all circRNAs with circular junction counts ≥ 1, 2, …,6. The relative circRNA expression levels were evaluated by the ratio of total circular junction counts to total gene counts.

### Characteristics of the circRNAs

The identified circRNAs were classified into three types, namely, exonic, intronic, and intergenic, according to their predefined type in CIRI. Notably, a circRNA with one end located in an intergenic or intronic region was categorized as “intergenic” or “intronic” regardless of where the other end was located.

The putative circRNA primary structures were predicted based on human gene annotations (GENCODE v19). Although exons can be removed and introns can be retained during circRNA formation, we considered only exons enclosed by splice sites. The lengths of the circRNAs were calculated by the sum of the exons, and the intron length was added only if it was enclosed by the back-spliced sites.

### Normalization and evaluation of covariates

The voom [20] normalization scales read each circular junction by total read counts across all circular junction sites in the SCZ and control samples and transforms it to the logarithm (base 2). We considered technical (institution, library preparation batch, postmortem interval (PMI), RNA integrity number (RIN), and RIN^2^) and biological (age of death (AOD), ethnicity, gender, SCZ) factors as covariates in voom normalization. We used the limma [21] package (version 3.34.5) to detect DE circRNAs among these covariates. The RIN- and RIN^2^-related circRNAs were merged.

Random sampling analysis was also employed to detect SCZ-associated DE circRNAs. Briefly, 50 or 100 SCZ cases were selected from all cases by stochastic, as well as the equivalent number for controls. SCZ-associated DE circRNAs were detected as described above. The procedure was repeated for 1000 times, and circRNAs reported to be DE in more than 50 random sampling were identified as SCZ-associated DE circRNAs.

For disease-specific expressed circRNA analysis, circRNAs expressed (circular junction counts ≥ 1 in more than half of individuals and average ratio ≥ 0.05) in either SCZ or control subjects were selected, and the same method described above was used to detect differentially expressed circRNAs in SCZ.

Raw gene counts were downloaded from the CMC database. We filtered out all genes with low expression in SCZ and control samples, leading to 16,422 genes remaining with at least 1 gCPM (gene counts per million total gene reads) in at least 50% of the individuals. Then, we used the same method to normalize gene expression and detect covariate-associated genes.

### Needed sample size to detect DE circRNAs or genes

Effect size of each circRNA or gene was estimated by |log_2_(*fold change*)|/*sd*, whereas *fold change* is calculated by the ratio of average expression level in SCZ to control, and *sd* is the standard deviation of all samples expression quantified by log_2_(circCPM) for circRNAs or log_2_(gCPM) for genes. Cutoff for significance level was estimated by 0.05/#(circRNAs or genes tested). The needed sample size was estimated by using pwr.t.test function of package pwr in R with the following parameters: *d* = *maximal effect size*, sig.level = *cutoff*, power = 0.8, type = “two.sample”, and alternative = “two.sided”.

### circQTL analysis

We first used voom to normalize all 589 samples using the above method but considered all disease stages as covariates, including SCZ, control, and AFF. Next, 465 genetically inferred Caucasians [11] remained, and the covariate effects were regressed out based on the weight of voom in a weighted linear regression.

The correlations between genotypes and covariate-adjusted circRNA expression, i.e., circRNA expression quantitative loci (circQTLs), were tested using Matrix eQTL [28] with the additive linear model on the imputed genotype dosages (only SNPs in autosomal regions were included in this study). SNPs between the 100-kb region upstream of the 5′ back-spliced site and the 100-kb region downstream of the 3′ back-spliced site were included for *cis*-regulation. Similar to a previous study [41], circQTL-containing circRNAs were identified by a permutation procedure that corrects for the multiple hypothesis effect of many variants in LD. Briefly, a total of 10,000 permutations were performed by randomizing sample labels for the circRNA expression matrix. The minimal *P* value (min*P*) for each circRNA in every permutation was collected to derive empirical *P* values (emp*P*) of uncorrected circQTLs for each circRNA. For each uncorrected circQTL *P* value (*P*_circQTL_), the emp*P* can be estimated by the following formula:

$$ \mathrm{emp}P=\frac{1+{\sum}_{n=1}^{10000}\#\left(\min {P}_n<{P}_{\mathrm{circQTL}}\right)}{10001} $$

min*P*_*n*_ represents the min*P* in the 10,000 permutations for the circRNA. *Q* values were calculated using Storey’s approach [29] based on the distribution of minimal emp*P* of all circRNAs. circRNAs with *q* values less than 0.05 were identified as circQTL-containing circRNAs. For each circQTL-containing circRNA, a emp*P* equivalent to a *q* value of 0.05 was deemed the permutation threshold to identify circQTL SNPs.

When measuring the circRNA expression level by the circular ratio, we performed multiple linear regression to regress out covariates and detect correlations between genotypes and the normalized circular ratio.

### Analysis of the characteristics of circQTL SNPs

The most significant SNP for each circQTL-containing circRNA was selected to study the circRNA characteristics. Linked SNPs were pruned with the reference from the 1000 Genomes Phase 1 genotype data [51] using the PLINK [52] -indep-pairwise module with the following parameters: --indep-pairwise 50 5 0.5. SNPs with minimal uncorrected *P* values larger than 0.05 were selected as negative controls, i.e., non-circQTL SNPs, after LD-based pruning and MAF distribution correction.

The max-circQTL and non-circQTL SNPs were classified into the following categories: splice acceptor, splice donor, start lost, stop gained, stop lost, missense, splice region, synonymous, untranslated region 5′ (5′-UTR), 3′-UTR, noncoding transcript exon, intron, upstream gene, downstream gene, and intergenic variant, using Variant Effect Predictor (VEP) [53] with the -most_severe parameter. The categories start lost, stop gained, and stop lost were removed due to low counts. In addition, the splice acceptor and splice donor categories were combined into one splicing category, i.e., canonical splice site. Finally, 11 types were retained for enrichment analysis by a two-tailed Fisher’s exact test.

### Detecting reverse complementary sequences (RCSs)

We detected RCSs according to a previous study [54]. Briefly, flanking introns of an exonic circRNA were aligned to each other using BLAST [55] (version 2.8.0+) with the parameters –task blastn –word_size 11 –strand minus. Potential RCSs were identified if the bitscores of alignments exceeded 100.

### Datasets of disease-related GWAS SNPs

The list of SNPs associated with various human traits was downloaded from the GWAS Catalog [34] (http://www.ebi.ac.uk/gwas, the gwas_catalog_v1.0.1 file, accessed Feb 2018). Significant disease-related GWAS SNPs (*P* ≤ 5 × 10^−8^) were extracted using the ontoCAT [56] package of R if they were associated with the child terms of “EFO_0000408: disease.” We performed a one-tailed Fisher’s exact test for the following diseases: (1) all human diseases (EFO_0000408: disease); and (2) 21 individual diseases with GWAS SNPs larger than 90, including age-related macular degeneration, allergy, Alzheimer’s disease, androgenetic alopecia, asthma, atrial fibrillation, autism, bipolar disorder, breast carcinoma, coronary artery disease, coronary heart disease, inflammatory bowel disease, lung carcinoma, Parkinson’s disease, prostate carcinoma, psoriasis, rheumatoid arthritis, schizophrenia, systemic lupus erythematosus, type I diabetes mellitus, and type II diabetes mellitus. SNPs linked (*r*^2^ > 0.6 calculated by PLINK) to these GWAS SNPs were also included in the enrichment analysis.

## Additional files

Additional file 1: **Table S1.** List of Ribo-zero RNA-seq data from ENCODE Project Consortium used in this study. (XLSX 16 kb)

Additional file 2: **Figure S1.** Relative circRNA expression level in 14 cell lines, 29 tissues, and DLPFC. **Figure S2.** The Sanger sequencing results for circRNAs from AKT3, HOOK3, and MDGA2. **Figure S3.** Distributions of the exon numbers (A) and lengths (B) of exonic circRNAs. **Figure S4.** Pie charts indicated the fraction of circRNAs produced from one host gene. **Figure S5.** LIB-associated circRNAs show higher expression levels and shorter median lengths. **Figure S6.** Comparison of distributions of effect size of circRNAs (red line) and genes (blue line) in detecting SCZ-control differential expression in CMC dataset. (DOCX 1163 kb)

Additional file 3: **Table S2.** List of 10,559 circRNAs. (XLSX 1499 kb)

Additional file 4: **Table S3.** Details of RT-PCR primers for validation of 13 selected circRNAs. (XLSX 10 kb)

Additional file 5: **Table S4.** Results of the gene-set enrichment analysis of host genes. (XLSX 18 kb)

Additional file 6: **Table S5.** List of circRNAs associated with age of death at an FDR < 0.05. (XLSX 30 kb)

Additional file 7: **Table S6.** List of 196,255 circQTLs. (XLSX 14566 kb)

Additional file 8: **Table S7.** The detail of circQTL SNPs located in the splicing site. (XLSX 17 kb)

Additional file 9: **Table S8.** List of circQTL SNPs linked to GWAS signals in the NHGRI GWAS Catalog. Each index SNP is significantly associated with disease (*P* ≤ 5 × 10^−8^) in GWAS. circQTL SNPs in high linkage disequilibrium (*r*^2^ > 0.8) with index SNPs were extracted. (XLSX 290 kb)

Additional file 10: In-house scripts for circRNA analysis. Scripts of RNA-seq, circQTL, and statistical analysis in this study. (ZIP 56 kb)

## Abbreviations

AFF: Affective/mood disorder
AGA: Androgenetic alopecia
AOD: Age of death
BC: Breast carcinoma
BP: Bipolar disorder
CAD: Coronary artery disease
CDS: Coding sequence
circCPM: Circular junction counts per million circular junction reads
circQTL: Circular RNA quantitative trait loci
circRNA: Circular RNA
CMC: CommonMind Consortium
DE: Differentially expressed
DLPFC: Dorsolateral prefrontal cortex
emp*P*: Empirical *P* value
eQTL: Expression quantitative trait loci
FDR: False discovery rate
FET: Fisher’s exact test
gCPM: Gene counts per million gene reads
GWAS: Genome-wide association studies
IBD: Inflammatory bowel disease
LIB: Library batch
lincRNA: Long intergenic noncoding RNA
MAF: Minor allele frequency
max-circQTL: The most significant circQTL for each circRNA
min*P*: Minimal *P* value
OR: Odds ratio
PMI: Postmortem interval
QTL: Quantitative trait loci
RCS: Reverse complementary sequence
RIN: RNA Integrity number
SCZ: Schizophrenia
sQTL: Splicing quantitative trait loci
T2DM: Type II diabetes mellitus
UTR: Untranslated region
