# Supplementary material for: Detection of circular RNA expression and related quantitative trait loci in the human dorsolateral prefrontal cortex
Source: Genome Biol. 2019 May 20;20:99. doi: 10.1186/s13059-019-1701-8 (PMC6528256; doi:10.1186/s13059-019-1701-8)
Supplement: Supplementary file 2 — Figure S1. Relative circRNA expression level in 14 cell lines, 29 tissues, and DLPFC. Figure S2. The Sanger sequencing results for circRNAs from AKT3, HOOK3, and MDGA2. Figure S3. Distributions of the exon numbers (A) and lengths (B) of exonic circRNAs. Figure S4. Pie charts indicated the fraction of circRNAs produced from one host gene. Figure S5. LIB-associated circRNAs show higher expression levels and shorter median lengths. Figure S6. Comparison of distributions of effect size of circRNAs (red line) and genes (blue line) in detecting SCZ-control differential expression in CMC dataset. (DOCX 1163 kb) [file 13059_2019_1701_MOESM2_ESM.docx]

**Figure S1**


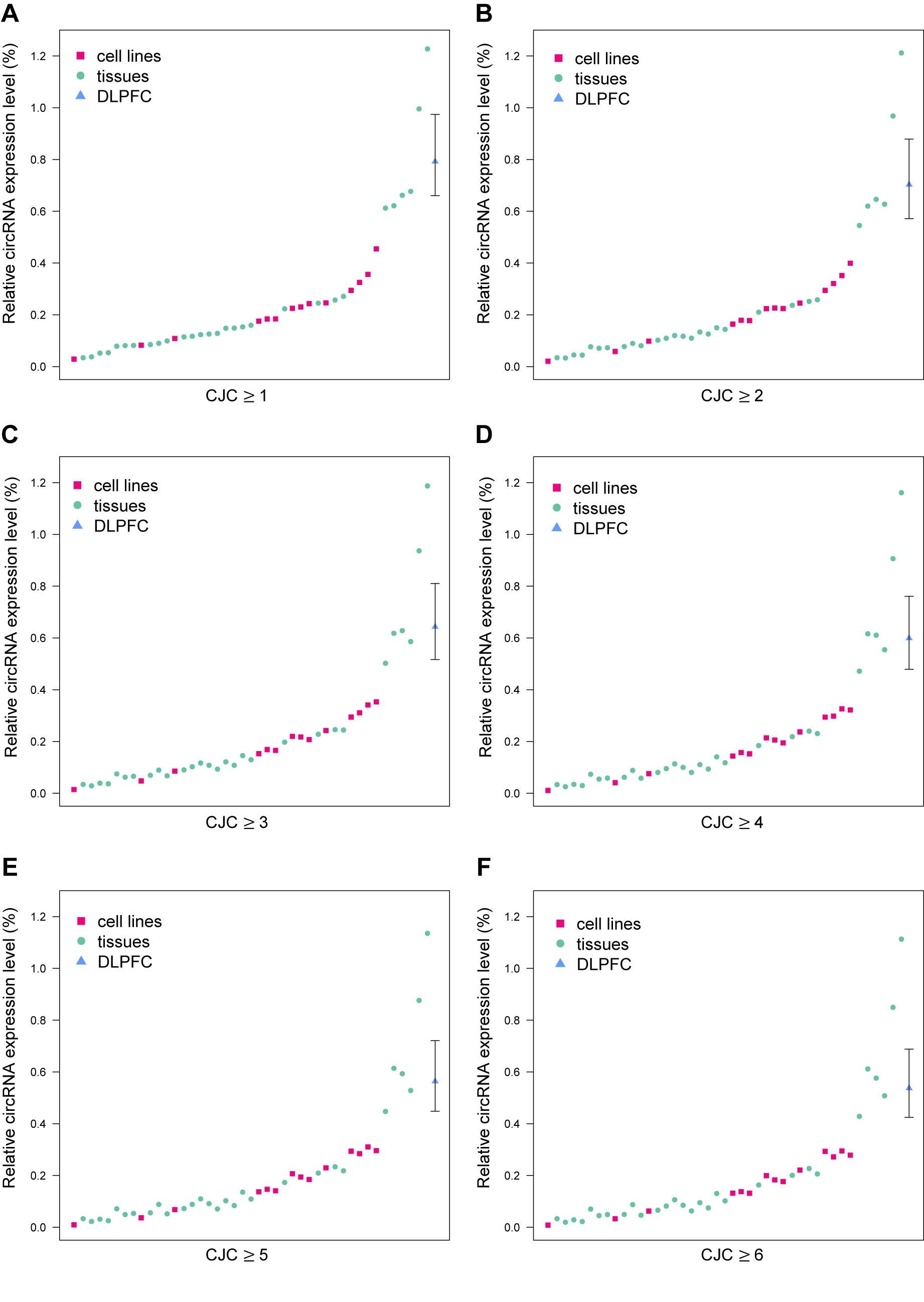


**Figure S1. Relative circRNA expression level in 14 cell lines, 29 tissues, and DLPFC.** The circRNA junction counts (CJC) of circRNAs with CJC ≥1 (A), 2 (B), …, 6 (F) were summed as total CJC. Relative circRNA expression level were evaluated by the ratio of total CJC to total gene counts. Points from left to right represent stomach, dendritic cell, Peyer's patch, heart left ventricle, right lobe of liver, adrenal gland, transverse colon, gastrocnemius medialis, K562, suprapubic skin, ascending aorta, omental fat pad, haoec_cell_total, subcutaneous adipose, body of pancreas, lower leg skin, vagina, spleen, breast epithelium, upper lobe of left lung, esophagus squamous epithelium, right atrium auricular region, cd34_cell_total, hmepc_cell_total, hsavec_cell_total, esophagus muscularis mucosa, hvmf_cell_total, hmscat010_cell_total, imr_cell_total, tibial nerve, hpiepc_cell_total, gastroesophageal sphincter, thyroid gland, haoaf609_cell_total, hch001_cell_total, hfdpc033_cell_total, hmncpb_cell_total, cerebellum, uterus, ovary, diencephalon, sigmoid colon, and frontal cortex, respectively. For DLPFC, the center blue triangle denotes the median of 589 samples, the limits are the interquartile range.

**Figure S2**


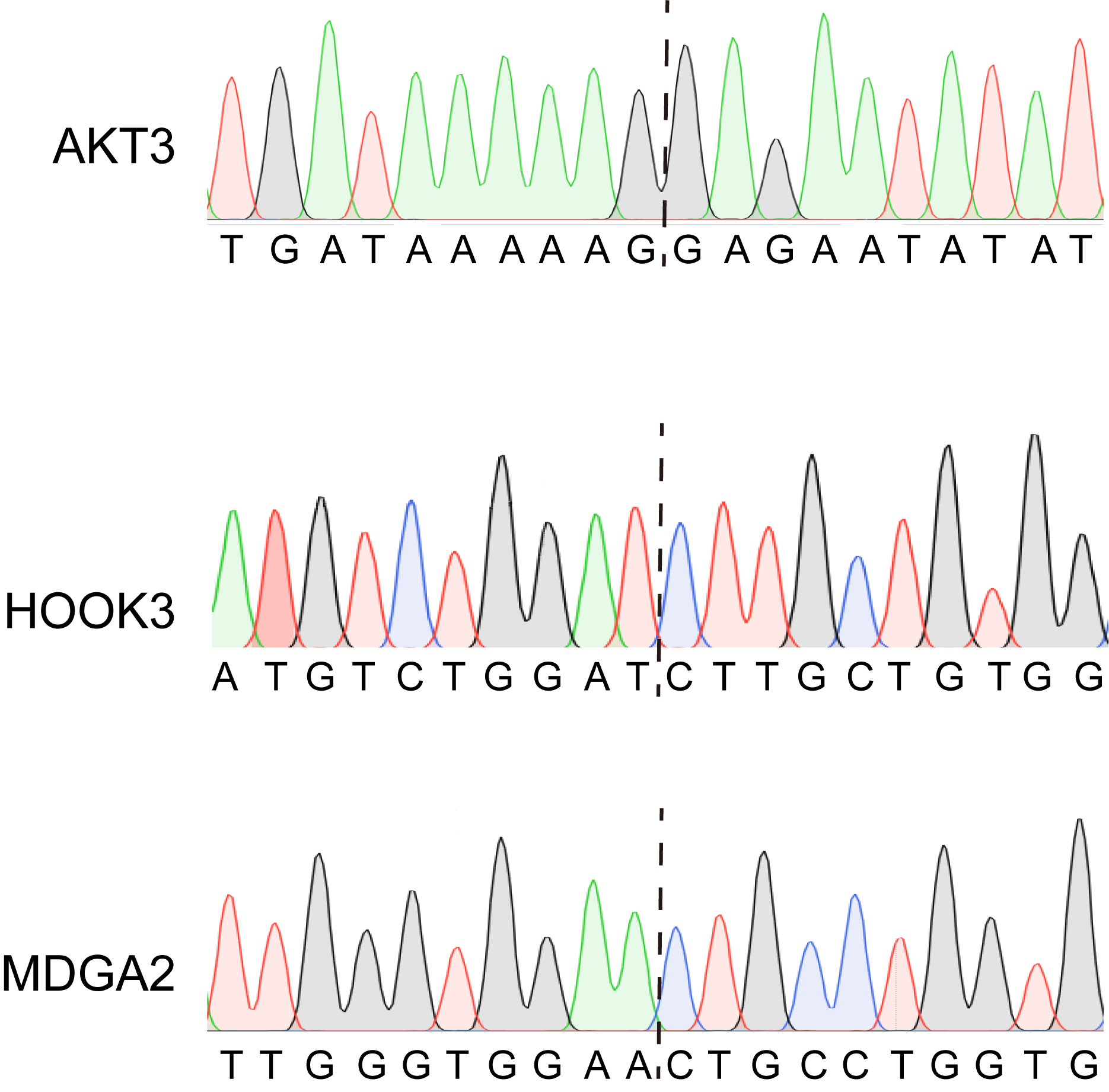


**Figure S2. The Sanger sequencing results for circRNAs from AKT3, HOOK3, and MDGA2.** Dashed line indicates the circular junction site.

**Figure S3**


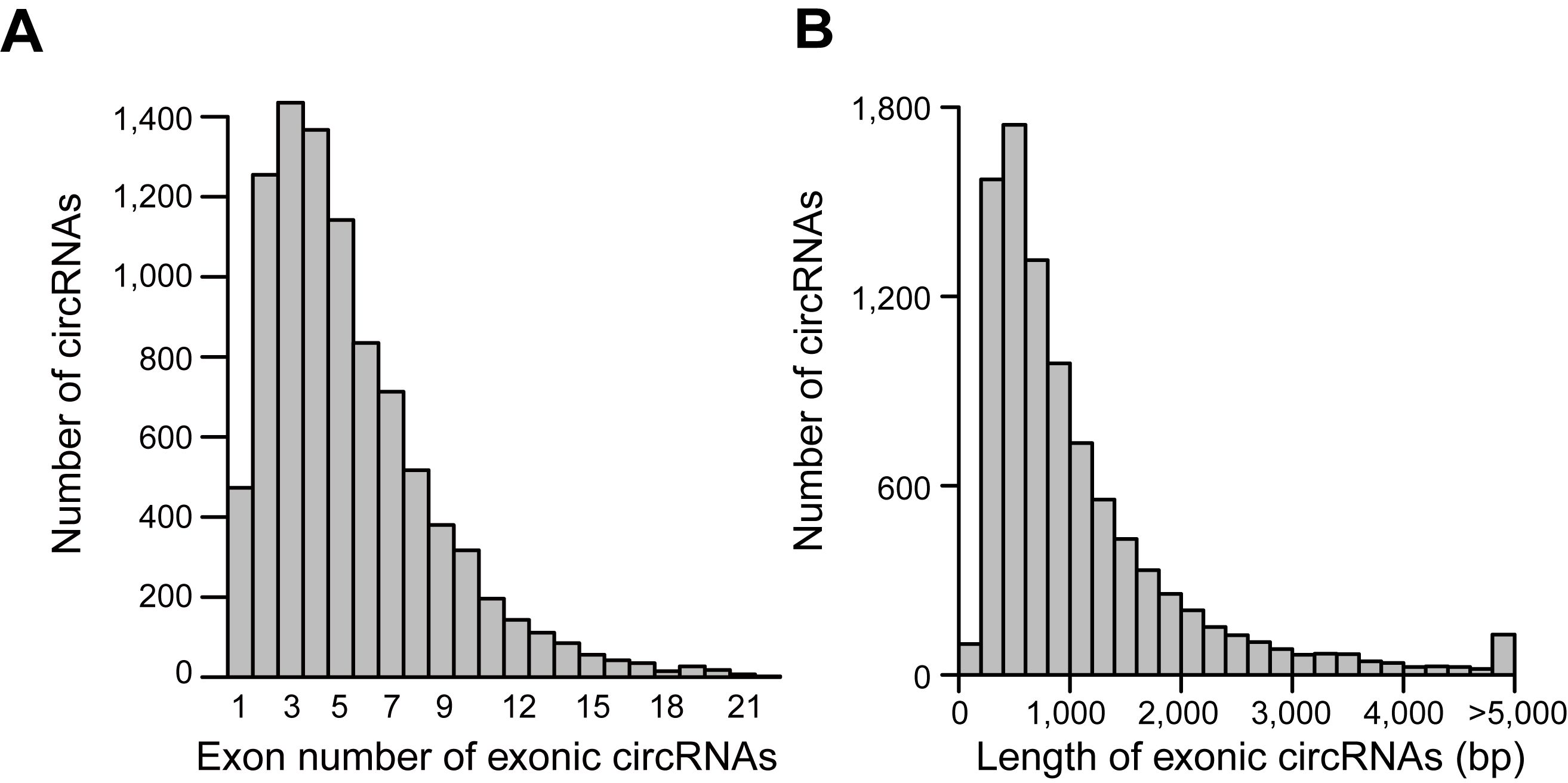


**Figure S3. Distributions of the exon numbers (A) and lengths (B) of exonic circRNAs.**

**Figure S4**


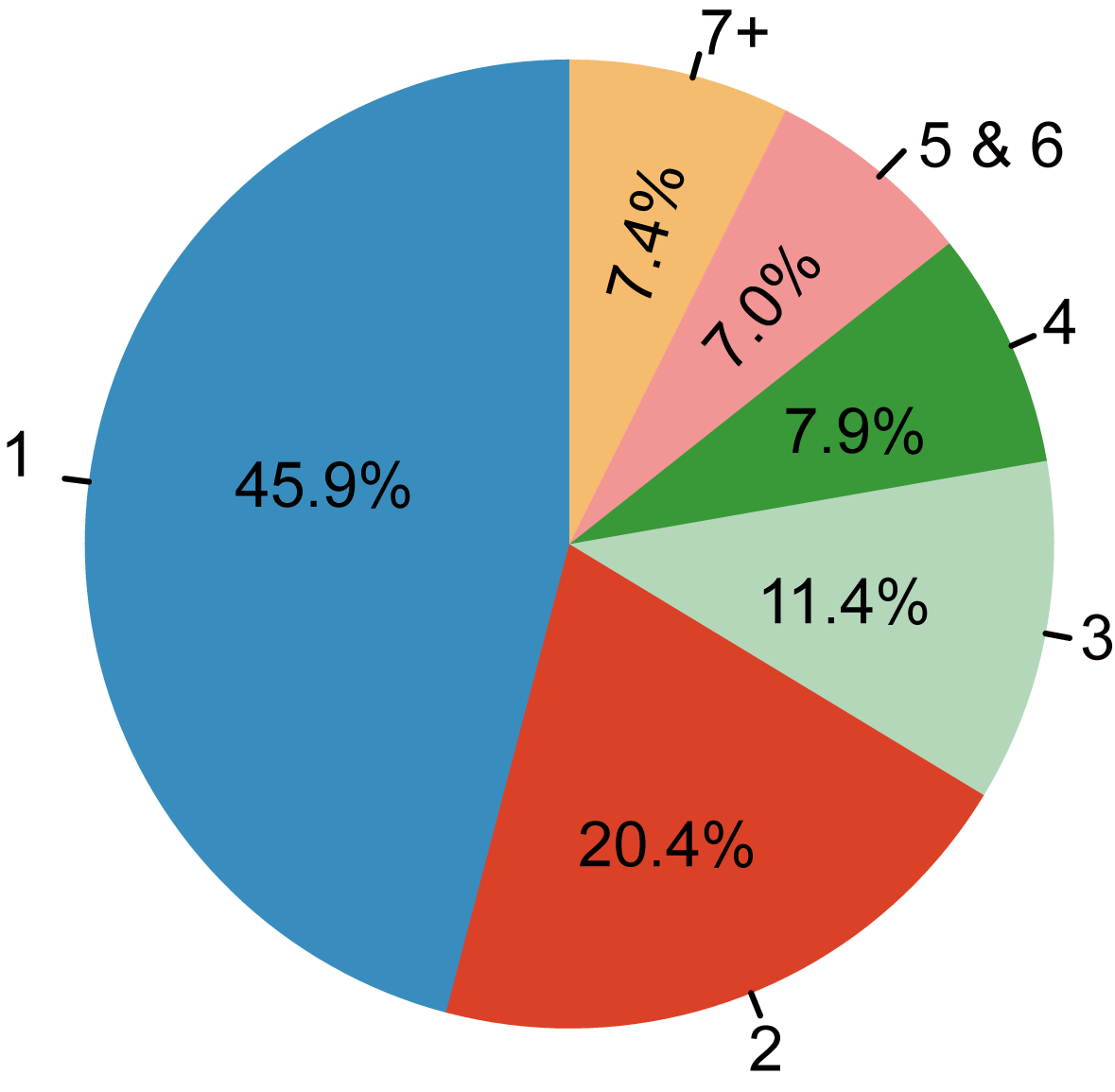


**Figure S4. Pie charts indicated the fraction of circRNAs produced from one host gene.**

**Figure S5**


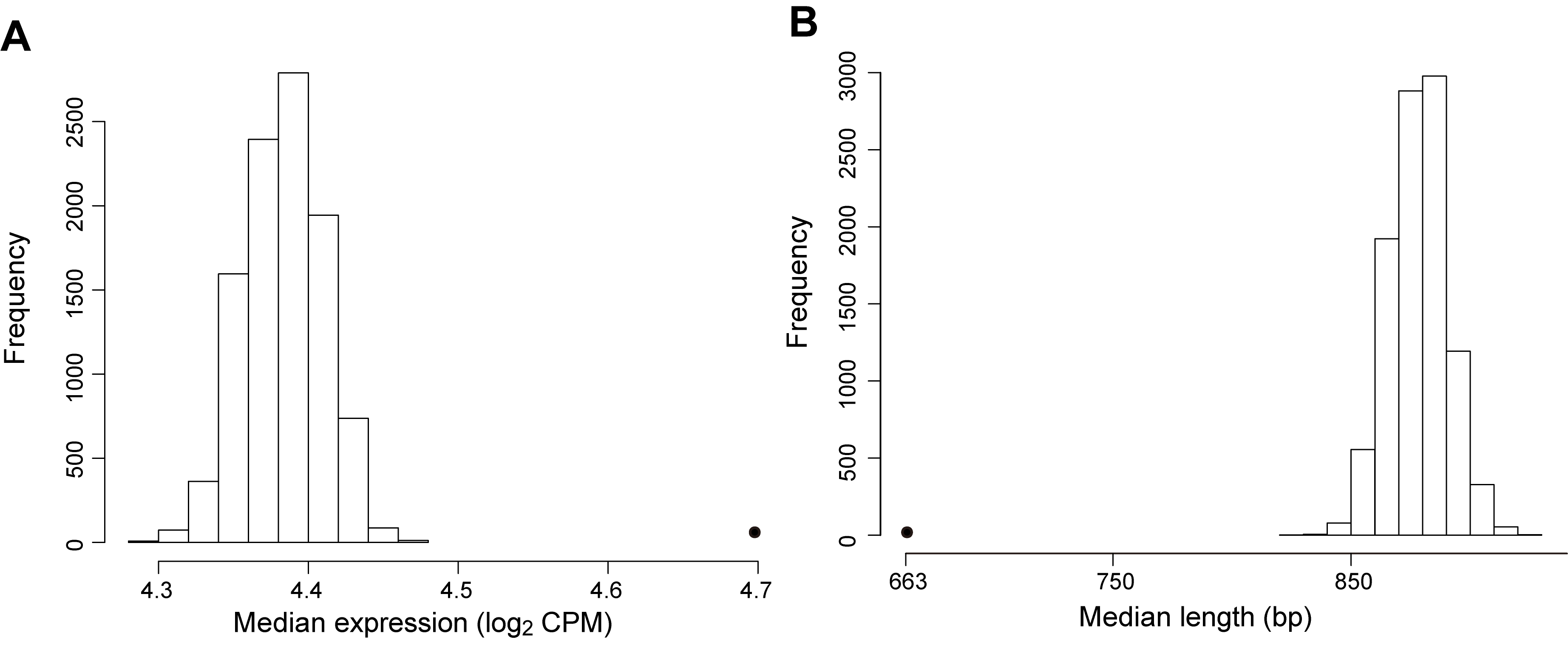


**Figure S5. LIB-associated circRNAs show higher expression levels and shorter median lengths. (A)** Distribution of 10,000 median expressions of 4,143 randomly sampled circRNAs. The black point represents the median expression levels of LIB-associated circRNAs. **(B)** Distribution of 10,000 median lengths of 4,143 randomly sampled circRNAs. The black point represents the median length of LIB-associated circRNAs.

**Figure S6**


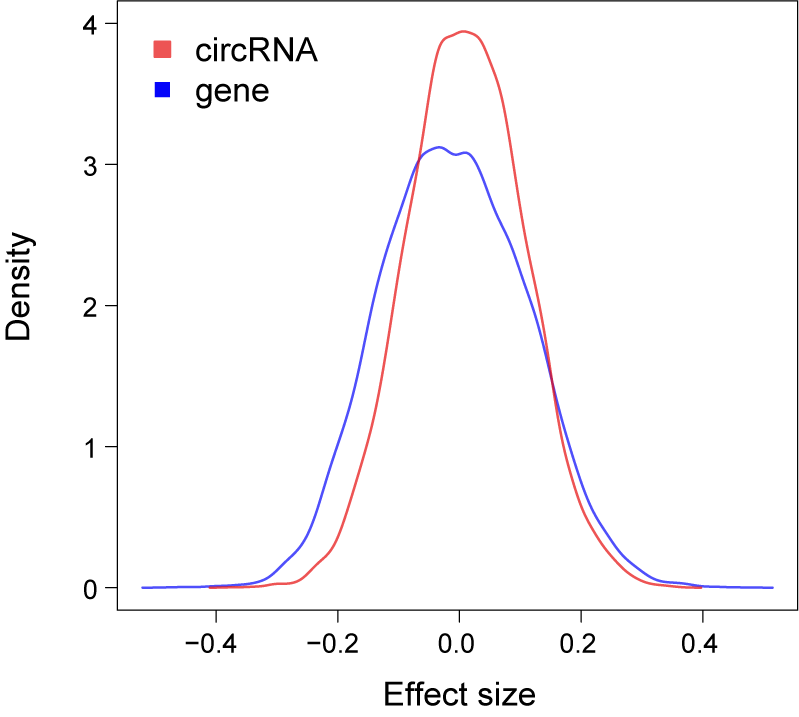


**Figure S6. Comparison of distributions of effect size of circRNAs (red line) and genes (blue line) in detecting SCZ-control differential expression in CMC dataset.**
